# Sequence specificity incompletely defines the genome-wide occupancy of Myc

**DOI:** 10.1186/s13059-014-0482-3

**Published:** 2014-10-07

**Authors:** Jiannan Guo, Tiandao Li, Joshua Schipper, Kyle A Nilson, Francis K Fordjour, Jeffrey J Cooper, Raluca Gordân, David H Price

**Affiliations:** Department of Biochemistry, University of Iowa, Iowa City, IA 52242 USA; Molecular and Cellular Biology Program, University of Iowa, Iowa City, IA 52242 USA; Institute for Genome Sciences and Policy, Duke University, Durham, NC 27708 USA; The Genome Institute, Washington University in St. Louis, St. Louis, MO 63108 USA

## Abstract

**Background:**

The Myc-Max heterodimer is a transcription factor that regulates expression of a large number of genes. Genome occupancy of Myc-Max is thought to be driven by Enhancer box (E-box) DNA elements, CACGTG or variants, to which the heterodimer binds *in vitro*.

**Results:**

By analyzing ChIP-Seq datasets, we demonstrate that the positions occupied by Myc-Max across the human genome correlate with the RNA polymerase II, Pol II, transcription machinery significantly better than with E-boxes. Metagene analyses show that in promoter regions, Myc is uniformly positioned about 100 bp upstream of essentially all promoter proximal paused polymerases with Max about 15 bp upstream of Myc. We re-evaluate the DNA binding properties of full length Myc-Max proteins. Electrophoretic mobility shift assay results demonstrate Myc-Max heterodimers display significant sequence preference, but have high affinity for any DNA. Quantification of the relative affinities of Myc-Max for all possible 8-mers using universal protein-binding microarray assays shows that sequences surrounding core 6-mers significantly affect binding. Compared to the *in vitro* sequence preferences, Myc-Max genomic occupancy measured by ChIP-Seq is largely, although not completely, independent of sequence specificity.

**Conclusions:**

We quantified the affinity of Myc-Max to all possible 8-mers and compared this with the sites of Myc binding across the human genome. Our results indicate that the genomic occupancy of Myc cannot be explained by its intrinsic DNA specificity and suggest that the transcription machinery and associated promoter accessibility play a predominant role in Myc recruitment.

**Electronic supplementary material:**

The online version of this article (doi:10.1186/s13059-014-0482-3) contains supplementary material, which is available to authorized users.

## Background

c-Myc was initially identified as a proto-oncoprotein and subsequently demonstrated to be a global regulator of transcription [1-6]. *In vitro*, the basic helix-loop-helix leucine zipper (bHLHZip) domain of Myc binds preferentially, albeit very weakly, to double stranded DNA containing the palindrome CACGTG and this is considered the canonical E-box [7,8]. Myc pairs with Max and the heterodimer binds to CACGTG with higher affinity [9-11]. A crystal structure of the bHLHZip domains of Myc-Max bound to DNA revealed that the two proteins interact through each protein’s bHLHZip domain and each make specific contacts with four bases [12]. These initial observations and a large number of studies on the effects of Myc on specific genes led to what is now the prevailing model that Myc, in conjunction with Max, binds to E-box sequences and subsequently regulates transcription by Pol II [1-4]. However, this model does not explain how specificity for Myc is achieved as there are a number of other bHLHZip protein family members that can bind to the same sequence [13,14].

Many studies attempting to identify Myc target genes found that the sets of genes regulated by Myc displayed great variation depending on the cell types and conditions used [15]. A significant advancement in understanding Myc function was achieved by two comprehensive studies from the Young and Levens labs [5,6]. Both used inducible systems to show that Myc, when switched on, utilized existing expression programs and globally amplified transcription leading to an increase in the majority of expressed mRNAs. One study concluded that induction of Myc in P493 cells led to increased binding by Myc-Max heterodimers at the E-box containing core promoter sequences of actively transcribed genes [5]. The other study using primary B cells treated with lipopolysaccharide to induce Myc expression found a only a ‘loose association’ of Myc with E-boxes due to the high frequency of random occurrence of degenerate E-box sequences [6].

Regulation of gene expression is controlled predominantly through the action of DNA-binding transcription factors that affect both initiation and elongation. A prominent feature of metazoan genomes is the promoter proximal paused Pol II that is found engaged in transcription about 30 to 80 bp downstream of the transcription start site (TSS) on most expressed genes [16-19]. The transition of these paused polymerases into productive elongation requires the kinase activity of the Positive Transcription Elongation Factor b, P-TEFb [16,20]. *MYC* was the first gene shown to be regulated by elongation [21] and Myc itself associates with P-TEFb [22-24] and causes an increase in productive elongation on targeted genes [25-27].

The mechanism of Myc regulation of transcription has been assumed to involve Myc-Max heterodimers binding to E-boxes near TSSs and then influencing the function of the transcription machinery. The discovery that Myc globally regulates essentially all expressed genes [5,6] hints that Myc recruitment may be more general. An earlier study showed that Myc occupancy primarily correlated with chromosomal loci with an ‘open conformation’ [28,29]. These regions are often occupied by the transcription machinery [19,30]. To test the basic assumption that Myc-Max heterodimers predominately occupy high affinity DNA elements in cells, we performed detailed analyses of available human ChIP-Seq datasets for Myc, Max, and Pol II and determined the relationship between sites of occupancy of Myc and Max and locations of high affinity DNA elements. Surprisingly, the global occupancy of Myc and Max strongly correlated with Pol II transcription machinery rather than with sequences that the heterodimer prefers *in vitro*.

## Results

### Genome occupancy of Myc and Max correlates with Pol II

**Figure 1:**
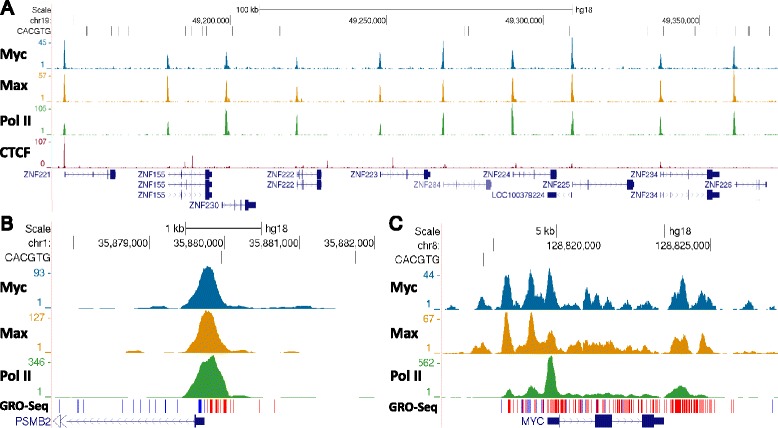
**Examples of Pol II, Myc, and Max occupancy.** Genome browser tracks show occupancy determined by ChIP-Seq for Pol II, Myc, Max, and CTCF over the indicated gene regions in HeLa cells. The positions of the canonical CACGTG E-boxes are indicated. Regions around **(A)** chromosome 19 containing 10 genes, **(B)** *PSMB2*, and **(C)** *MYC* are shown. GRO-Seq data are for IMR90 cells from GSE13518 [34].

Using the UCSC Genome Browser [31] and ChIP-Seq datasets generated from HeLa cells [32] with antibodies stringently validated by the ENCODE project [33], occupancies of Myc and Max visually correlate with Pol II better than with the E-box element CACGTG. For example, a broad view of 10 genes across a 200 kb region shows almost identical patterns for Myc and Max and a high level of visual correlation with promoter proximal paused polymerases on each of the genes (Figure 1A). Many genes exhibit divergent transcription as indicated by GRO-Seq [34] that can result in paused Pol II in both orientations. A closer view of one such gene demonstrates that Myc and Max reside in a position between the two peaks of Pol II (Figure 1B). It is important to remember that the position of the immunoprecipitated factor is not indicated by the envelope of mapped DNA fragments, but rather by the peak of that envelope. Visual analysis of highly expressed genes, exemplified by *MYC*, provides further evidence that Myc and Max occupancy is tied to Pol II, including polymerases within the transcribed regions and downstream of the Poly(A) addition site (Figure 1C). For the three regions shown there is almost no correlation of Myc or Max with the canonical CACGTG E-box (Figure 1). In comparison, distributions of CTCF [35] and a number of other DNA-binding transcription factors (Additional file 1: Figure S1) are distinct from Myc, Max, and Pol II. When entire datasets were analyzed, genomic regions occupied by Myc exhibited a much more significant overlap with Pol II ChIP-Seq peaks than with the E-box element CACGTG (Fisher’s exact test: *P* value < 10^-300^ vs. 4.5 × 10^-7^).

**Figure 2:**
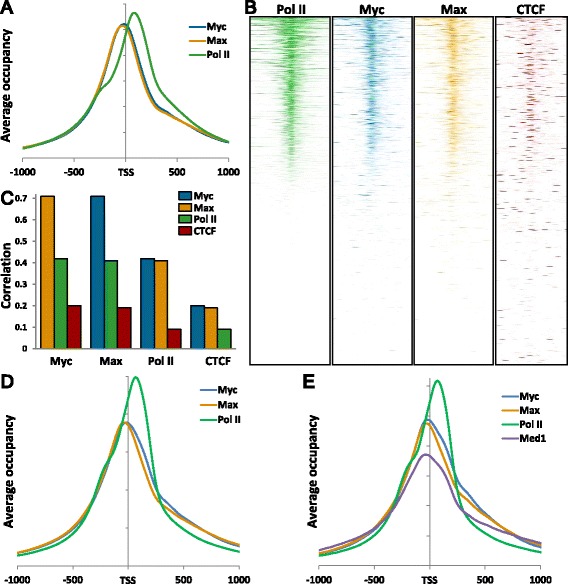
**Correlation of Myc and Max with Pol II occupancy. (A)** Metagene analysis showing the average of 20,886 genes. **(B)** High resolution heatmaps of the same genes rank-ordered by Pol II occupancy. The region shown is from -2 kb to +2 kb around the TSS. **(C)** Correlation of the occupancy of the indicated proteins. **(D)** Metagene analyses of Myc, Max, and Pol II ChIP-Seq datasets from eight different cell lines (HeLa, GM12878, K562, H128, H2171, MM1S, P493, and U87). **(E)** Metagene analysis of Myc, Max, Med1, and Pol II ChIP-Seq datasets from four different cell lines (H2171, MM1S, P493, and U87). Average occupancies of regions from -1,000 to +1,000 bp around the TSS are shown.

Several straightforward bioinformatic tools were used to obtain a global view of the correlation of Myc and Max compared to Pol II and CTCF. The average occupancy around the TSS of 20,886 genes in HeLa cells was calculated and plotted. Promoter proximal paused Pol II peaked on average 83 bp downstream of the TSS. Myc and Max on average peaked upstream of the TSS at -20 and -35, respectively (Figure 2A). Myc and Max also exhibited a slope transition at around +300 which has been previously noted for Pol II, the Med1 subunit of Mediator, and other transcription factors [36,37]. High resolution heatmaps were generated to assess the uniformity of these distributions in the 4 kb region centered on the TSSs across the same gene set (Figure 2B). Genes were ranked by the amount of Pol II in all four heatmaps. The patterns for Myc and Max occupancy are essentially identical and they closely match the occupancy pattern for Pol II, but not CTCF. These results indicate that Myc and Max are found about 100 bp upstream of the promoter proximal paused Pol II on most of the genes occupied by Pol II. In addition, Myc and Max were also positioned very closely with Pol II in enhancer regions (Additional file 1: Figure S1C).

These ChIP-Seq datasets were also compared using an algorithm that measures the similarity of peak positions and heights in any two datasets (Figure 2C). A value of 0 means there is no overlap of the signals at any position and 1 indicates the datasets are identical. Myc and Max most closely correlate with each other, as expected. Importantly, the second highest genome-wide correlation for both Myc and Max was Pol II. The correlation of Myc with Pol II would not be expected to be as high as its correlation with Max because of the approximately 100 bp offset of Myc (and Max) from the peaks of promoter proximal paused Pol II. As expected, CTCF was the least well correlated with all datasets because it is bound by its CTC-containing motif mainly in intragenic regions [35]. The correlation analysis was extended to include Fos, Jun, and E2F1 and none of these factors correlated as well with Pol II as Myc and Max (Additional file 1: Figure S2).

We extended our analyses to eight human cell lines with Myc, Max, and Pol II ChIP-Seq datasets. All eight datasets were combined into a multi-genome metagene analysis and the results clearly indicated that on average, as was found in HeLa cells, Myc and Max were about 100 bp upstream of the promoter proximal paused Pol II and Myc is shifted downstream from Max (Figure 2D). Datasets for the Med1 subunit of Mediator were available for four of these cell lines and the multi-genome analysis displayed a similar distribution for Myc and Med1 including a downstream bulge over the promoter proximal paused Pol II (Figure 2E). These analyses strongly suggest that the Myc might be recruited to these genomic loci by the transcription machinery, with Mediator as a reasonable candidate.

### Under stoichiometric conditions with high concentrations of proteins and DNA, Myc-Max heterodimers display relaxed sequence specificity

**Figure 3:**
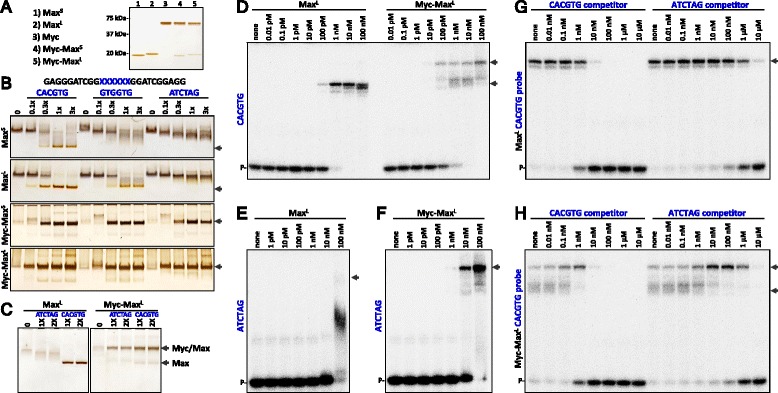
**Biochemical analysis of Myc and Max. (A)** SDS-PAGE of the indicated recombinant proteins that were expressed in *E. coli* and purified as described in Methods. **(B)** EMSA using native polyacrylamide gel electrophoresis with 200 ng of the indicated proteins (250 nM Max dimer and 125 nM Myc-Max) with 0, 0.1, 0.3, 1, or 3-fold molar excess of the indicated dsDNA. The gels were silver stained to show the mobility of the proteins. The arrows indicate protein-DNA complexes. **(C)** EMSA with simultaneous staining of Max^L^ and Myc-Max^L^. A total of 2.5 pmole of each protein (125 nM) per lane with two levels of the indicated DNA probes. Complexes containing indicated proteins are indicated with arrows. Note that in the Myc-Max prep some dissociation of Max has occurred leading to a low level of Max and Max-DNA species. **(D, E, F, G, and H)** EMSAs using 0.01 nM of the indicated radiolabeled probe (blue) with the indicated concentration of proteins and competitor DNAs.

Because of the low correlation between Myc-Max genome occupancy and CACGTG sequences, we re-examined the DNA binding properties of the Myc and Max proteins. Full length versions of Myc and two isoforms of Max, Max^S^ and Max^L^, were expressed in *E. coli* and purified to homogeneity (Figure 3A). The two Max isoforms were also individually mixed with Myc under denaturing conditions, allowed to refold using a step dialysis protocol, and then purified to obtain native, homogeneous heterodimers of Myc-Max^S^ and Myc-Max^L^ (Figure 3A). Electrophoretic mobility shift assays were carried out using three 26 bp dsDNA oligos that were identical except for the center 6 bps that contained the canonical CACGTG E-box, GTGGTG, or a completely unrelated sequence ATCTAG (Figure 3B). Native gels were silver stained to examine the shift in the position of 200 ng of protein. As expected, both homodimeric Max isoforms bound stoichiometrically to the CACGTG containing probe producing protein/DNA complexes that migrated further than the free proteins. Max^S^ displayed only very weak, transient binding to the other two probes while Max^L^ had reduced, but significant affinity for GTGGTG and low affinity for the ATCTAG probe (Figure 3B). Both Myc-Max complexes, regardless of Max isoform, produced a discrete protein DNA complex with the CACGTG probe. Surprisingly, both heterodimers bound stoichiometrically to the other two non-E-box probes (Figure 3B). Two individual studies assaying DNA binding with the same full-length proteins yielded identical shifting patterns [5,38]. The differences in the relative levels of staining of free and DNA-bound forms of Max versus Myc-Max was caused by differences in the staining (development time) of the four representative gels shown. When Max^L^ and Myc-Max^L^ were analyzed on the same gel they displayed similar staining levels and comparable increases in staining when bound to DNA (Figure 3C). It is important to understand that these EMSAs (Figure 3B and C) were carried out under stoichiometric conditions with high concentrations proteins and DNA. These conditions do not allow the determination of dissociation constants and, especially for Myc-Max, do not display the sequence specific differences in binding that are known to exist. Instead they show that Myc-Max can bind to any DNA sequence at the high, but not unreasonable concentration tested (125 nM). The Myc-Max-DNA complexes showed only a small change in mobility comparing to the free proteins. This could be due to a change in conformation of Myc-Max that leads to a lowering of the mobility like that seen for HEXIM1 bound to 7SK RNA [39].

Dissociation constants of the protein-DNA complexes were determined under the required non-stoichiometric conditions using 0.01 nM radiolabeled probe. Max^L^ and Myc-Max^L^ displayed tight binding to CACGTG (K_d_s of 0.4 nM and 0.1 nM, respectively) (Figure 3D). Max^L^ did not form a discrete complex with the ATCTAG probe with the concentrations of protein tested (K_d_ >1 μM), but instead gave only a smeary band below the position of a tightly bound complex (arrow) (Figure 3E). This is due to initial binding followed by release of the probe during the running of the gel. Myc-Max^L^ displayed significant affinity for the ATCTAG probe (K_d_ = 20 nM) (Figure 3F). Competition binding assays under these non-stoichiometric conditions demonstrated that CACGTG containing DNA was able to compete with the binding of Max^L^ and Myc-Max^L^ to the CACGTG probe (Figure 3G and H). At 1,000-fold higher concentration, the ATCTAG containing DNA was also able to compete for binding of both Max and Myc-Max to the CACGTG probe (Figure 3G and H). These results indicate that both Max and Myc-Max prefer to bind to the probe containing CACGTG as expected. In the stoichiometric assay described above, 125 nM Myc-Max but not 250 nM Max dimer formed discrete complexes with ATCTAG DNA. In the non-stoichiometric assay, Myc-Max displayed significantly higher affinity for the ATCTAG probe than Max and this difference was seen at 10 and 100 nM protein (Figure 3F). In the competition assay (1 nM protein) the difference between Myc-Max and Max was not seen. The concentration dependent change in the relative binding of Myc-Max and Max to non-specific DNA we observed could be related to the different on and off rates for the two proteins [40]. From all of the *in vitro* binding studies shown so far, we conclude that Myc-Max demonstrates a sequence preference, but that it also has significant affinity for DNA lacking a canonical E-box.

### Determination of the complete sequence preference for Myc-Max and comparison with occupancy in cells

**Figure 4:**
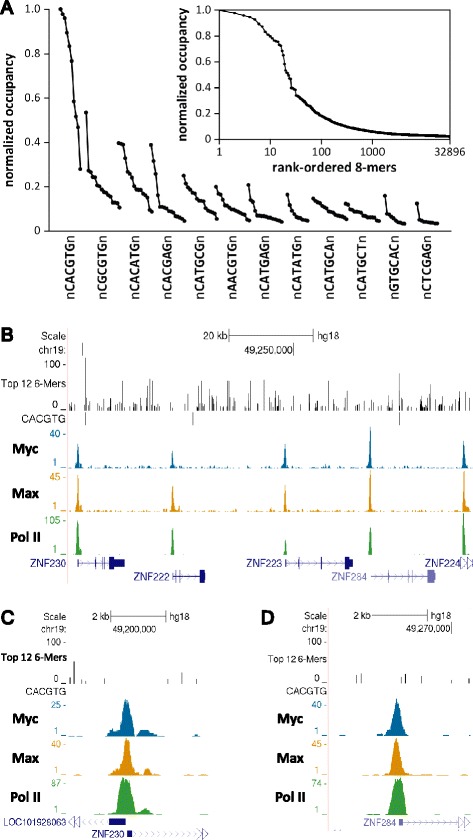
**Binding of Myc to all possible 8-mers and comparison with genomic occupancy. (A)** Fluorescent signal generated by Myc *in vitro* binding with an array containing all possible 8-mers was normalized. Twelve core 6-mer sequences with the highest *in vitro* occupancy are shown. The relative affinity of all 8-mers for each 6-mer is shown (10 points if the 6-mer is a palindrome or 16 if it is not). The inset shows the sorted *in vitro* binding signal for all possible 8-mers. **(B)** Genome browser view of a region on chromosome 19 comparing Myc, Max, and Pol II occupancy with the distribution of the top 12 6-mers (from A). The height of each 6-mer peak is equal to its relative *in vitro* occupancy (shown as percent). **(C, D)** Zoomed in views of two regions shown in (B) that demonstrate the lack of correlation of Myc and Max occupancy with the intrinsic affinity for the underlying DNA determined *in vitro*.

In our first attempts at trying to compare the *in vivo* occupancy of Myc and Max to the location of E-boxes, we ran into difficulty because of the existence of a large number of reported non-canonical E-boxes. Without quantification of the relative affinity of Myc-Max for all these sites it was difficult to correlate them with *in vivo* occupancy. Because of this, protein-binding microarray (PBM) assays using ‘all 10-mer’ universal array designs [41,42] were used to quantify the relative occupancies of the Myc-Max^L^ heterodimer and the Max^L^ homodimer across all possible 8 bp sequences (that is, 8-mers). After normalization, relative Myc-Max occupancy for each of the 32,896 8-mers exhibited a 56-fold range, from 0.018 to 1 (Figure 4A, inset). Although the method is very different from the EMSA assay described above, the PBM results also reflect the relaxed sequence preferences of Myc-Max. Most of the sequences containing CACGTG had high occupancy, but flanking bases had a significant influence (Figure 4A). In addition, we found several E-box variants and other core 6-mers with relatively high Myc-Max occupancy. The top 12 core 6-mers and the effect of the flanking bases are shown in Figure 4A. Like the canonical CACGTG core, Myc-Max occupancy of the other core 6-mers was significantly affected by flanking bases.

The problem of not knowing the relative affinity of Myc-Max for the previously proposed non-canonical E-boxes was resolved by the PBM assays so we used that information to examine the role intrinsic DNA affinity plays in the occupancy of the heterodimer in cells. A genome browser track comprising the location and relative *in vitro* occupancy (percent of the top binding site) of each of the top 12 6-mers was generated that graphically displays the range of intrinsic affinities across the genome (Figure 4B). This is an improvement compared to just marking canonical and non-canonical E-boxes without regard to relative affinities of the different sites. Visual comparison of the occupancy of Myc, Max, and Pol II in HeLa cells to the accurate distribution of intrinsic affinities does not provide evidence for a strong correlation between intrinsic affinity and occupancy in cells (Figure 4B). Closer inspection revealed that strong binding sites were not occupied and Myc and Max were found in regions that did not have any of the top 12 6-mer sites (Figure 4C and D).

**Figure 5:**
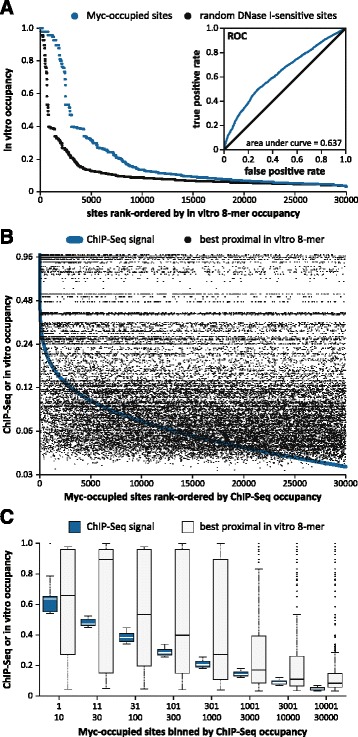
**Comparison of Myc ChIP-Seq occupancy with** ***in vitro*** **binding affinities. (A)** The top 30,000 sites occupied by Myc (blue) were rank-ordered and scored by the *in vitro* occupancy of the best 8-mer in a 100 bp window (y-axis). This was repeated at 30,000 random locations of DNase I-sensitivity (black) and the results were directly compared by ROC analysis (inset). **(B)** The top 30,000 sites occupied by Myc were rank-ordered by ChIP-Seq signal and scored logarithmically by either normalized ChIP-Seq signal (blue line) or the *in vitro* occupancy of the best 8-mer in a 100 bp window (black dots). **(C)** The data in **(B)** are presented using a default R boxplot (box: 1st to 3rd quartile, line: median, whiskers: 1.5 × interquartile range beyond the box, outliers are stacked) with ChIP-Seq signal in blue and *in vitro* 8-mers in grey.

Several analyses were performed to compute the correlation between the 8-mer sequence preferences determined by PBM and the actual genomic occupancy of Myc, as measured by ChIP-Seq. The ChIP-Seq Peak algorithm [36] was used to determine the genomic location of each of the top 30,000 Myc peaks in HeLa cells. A 100 bp interval surrounding each peak was scanned to find the 8-mer with the highest possible *in vitro* occupancy and this score was assigned to each ChIP-Seq peak. These *in vitro* occupancy scores were normalized to 1, rank-ordered from highest to lowest values, and then plotted for all 30,000 peaks (Figure 5A, blue plot). Seventy-four percent of these Myc peaks were associated with low affinity 8-mers with *in vitro* occupancies below 0.2. To determine if the distribution of 8-mers around sites of Myc occupancy is different from what occurs by chance, the same analysis was performed on 30,000 100 bp regions randomly chosen from accessible DNA (DNase I sensitive regions [43]) (Figure 5A, black plot). The choice of DNase I sensitive regions as control sequences for this analysis is justified by the fact that 95% of the Myc peaks fall within such regions. Comparison of the two plots indicated that, as expected, genomic loci occupied by Myc contain more sites with high *in vitro* Myc occupancy compared to random accessible DNA regions (Wilcoxon rank-sum test: *P* value < 2.2 × 10^-16^). This enrichment is further shown by means of a receiver operating characteristic (ROC) curve (Figure 5A, inset). ROCs are commonly used in genomic analyses to assess whether a specific quantitative feature (here, *in vitro* Myc occupancy) can distinguish between two classes of sequences (here, ChIP-Seq peaks versus random accessible regions). Although the area under the ROC curve is better than expected by chance (0.637 vs. 0.5), the ROC analysis shows that the *in vitro* 8-mer occupancies cannot be used to accurately predict whether an accessible genomic region will be bound by Myc in cells. Here, the ROC plot shows that at a false positive rate of 0.1, the true positive rate is only 0.25. To make only 10% false positive predictions of Myc *in vivo* binding using the *in vitro* 8-mer scores, we would only be able to capture 25% of the true Myc ChIP-Seq peaks. This means that the vast majority of sites occupied by Myc are associated with low scoring 8-mers, as graphically indicated in Figure 5A.

To further assess whether the intrinsic binding specificity of Myc-Max determines its level of genomic occupancy in the cell, the same Myc sites were rank-ordered by their ChIP-Seq occupancy and compared to the signal of the best 8-mer within a 100 bp window around each peak. The Myc ChIP-Seq signal of the top 30,000 peaks varies about 30-fold (Figure 5B, blue line showing decreasing occupancy from left to right). Using the same x-axis, a second plot was generated that displays the relative affinity of the best 8-mer associated with each of these Myc peaks (Figure 5B, black dots). A slight preference for high affinity 8-mers is visible over the top 5,000 Myc peaks, but the overwhelming conclusion is that 8-mers with a wide range of *in vitro* occupancies are found around Myc peaks irrespective of the level of *in vivo* occupancy (Figure 5B). While a statistically significant correlation can be observed between Myc ChIP-Seq occupancy and *in vitro* 8-mer binding strength, this relationship is weak (Spearman correlation coefficient: ρ = 0.22, *P* value < 2.2 × 10^-16^). Had the cellular occupancy correlated well with the affinity for the underlying DNA sequences, there would have been a cloud of black dots clustered around the blue curve in Figure 5B and the Spearman correlation coefficient would have been close to 1. A plot of the same data after ChIP-Seq peaks were grouped into log-scaled bins provides a more detailed view of the high occupancy sites in cells that might be expected to correlate better with intrinsic DNA affinities. However, the huge range of *in vitro* occupancy scores is clearly found even for the highest occupancy sites (Figure 5C). All these analyses suggest that Myc occupancy is driven only to a small extent by its intrinsic sequence preference, and additional mechanisms are required to recruit Myc to its genomic binding locations in the cell.

### Genomic sites with higher relative levels of Max

**Figure 6:**
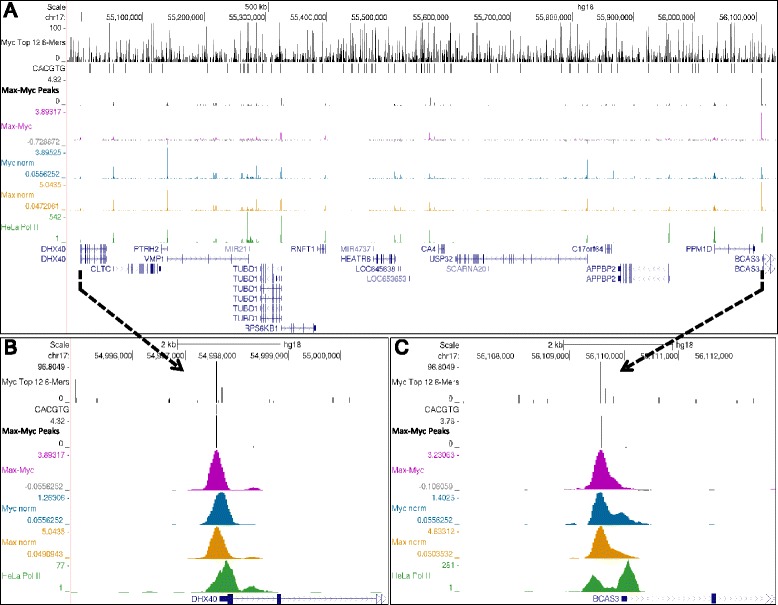
**Examples of sites with more Max than Myc.** Genome browser views of normalized Myc, Max, and ‘Max minus Myc’ occupancy and peaks generated by ChIP-Seq Peak. The distribution of the top 12 6-mers with their relative *in vitro* occupancies is also displayed. **(A)** A large region from chromosome 17. **(B, C)** Close-ups of the two regions with extra Max showing alignment with high scoring 6-mers.

Apart from associating with Myc, Max can form Max-Max homodimers or bind with Mad proteins to form Mad-Max heterodimers [44] and these can also bind E-box DNA sites [45]. We reasoned that such sites might have more Max than Myc. To identify these sites the HeLa Myc and Max datasets were normalized and a new track was generated in which the ChIP-Seq signal for Myc was subtracted from the signal for Max. Several thousand peaks with significant levels of extra Max were found. A representative region of chromosome 17, covering about 1 million bps that contains more than a dozen genes occupied by Pol II, Myc, and Max, is shown in Figure 6. The region contains about 20 peaks of Myc and Max and two of these sites have significant levels of extra Max. Both peaks of extra Max are on top of high scoring CACGTG sites (Figure 6B and C). Interestingly, the top 5,000 sites with extra Max (difference values greater than 0.5) were more tightly associated with high scoring 8-mers than were Myc sites (Additional file 1: Figure S3A) and had a more significant overlap with CACGTG than the Myc sites (Fisher’s exact test: *P* value <10^-300^ for extra Max sites vs. 4.5 × 10^-7^ for Myc sites). The top 1,487 peaks of extra Max (difference values greater than 1.0) were selected for further analysis (Additional file 2: Table S1). These sites were always close to peaks of Myc, Max, and Pol II, but only 417 of these peaks were within 250 bp of an annotated TSS. Gene Ontology (GO) analysis was performed on the associated genes, but no significant enrichment in any type of gene was uncovered. To determine if sites of extra Max might affect gene expression, the mRNA levels of those genes were compared to the mRNA levels of the top 12,000 expressed genes as determined by RNA-Seq. The RNA levels of 351 (of the 417) genes that were identifiable in the RNA-Seq dataset were distributed uniformly across the entire range of top 12,000 expressed genes covering more than three orders of magnitude in RNA levels (Additional file 1: Figure S3B). Thus, the sites with extra Max do not seem to be associated with any particular set of genes and do not correlate with the expression level of the genes they are associated with. Overall, sites with extra Max showed a stronger preference for E-box elements compared to Myc.

## Discussion

**Figure 7:**
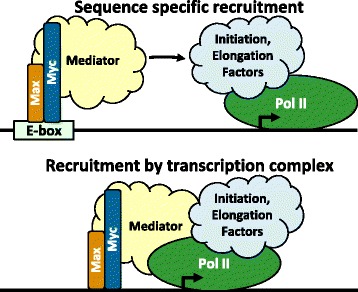
**Two models of Myc-Max recruitment.** The top panel illustrates the prevailing view of E-box recruitment of Myc-Max followed by interactions of Mediator and other factors that affect transcription. The bottom panel is an alternative view supported by the results presented here in which Myc-Max is recruited by the transcription machinery and bound with a relaxed sequence requirement to promoter DNA. We suggest that the Myc-Max occupancy of the promoter region helps keep the promoter free of nucleosomes and the resulting increased accessibility of the promoter is responsible for the amplification of gene expression caused by Myc.

The results presented here provide evidence supporting a perspective for Myc function where the transcription machinery rather than DNA sequence elements plays a major role in recruiting the Myc-Max heterodimer to genomic sites. Although other studies have found Myc near TSSs [5,6,35,46,47], previous models for Myc function (Figure 7, top panel) evoked recruitment of the heterodimer to E-box sequences as an initial step (see recent reviews [1,2]). However, we showed that sites of Myc occupancy were more highly correlated with Pol II rather than specific sequence elements. For sites near annotated TSSs, Myc was found about 100 bp upstream of the promoter proximal paused Pol II. We propose that for a large fraction of genes, the transcription machinery (which includes both initiation and elongation factors) is primarily responsible for recruitment of Myc-Max. The affinity of Myc-Max for DNA (K_d_ = 10^-8^ to 10^-10^ M, depending on the sequence) could then stabilize this interaction with specific sequence elements playing only a minor role (Figure 7, lower panel). The interaction(s) leading to Myc-Max occupancy is likely between the highly unstructured N-terminal transcription activation domain of Myc and factors in the transcription machinery. The model explains how Myc could influence entire transcription programs [5,6]. One possible mechanism for Myc function that is consistent with what we now know is that Myc could be recruited by Mediator or another factor which is associated with the promoter proximal paused Pol II and then bind with Max relatively non-specifically to the promoter DNA. This could help keep the promoter region free of nucleosomes and primed for preinitiation complex formation if the paused polymerase was released into productive elongation or terminated. This mechanism could explain how Myc leads to universal amplification of gene expression in that it would generally increase accessibility of promoters and, therefore, responsiveness to the signals that regulate transcription of specific genes.

Consideration of the size of the mammalian nucleus, the amount of Myc expressed, and the biochemical parameters of Myc-Max binding to DNA determined here leads to the interesting conclusion that almost all the Myc should be bound to DNA regardless of the influence of specific sequences. Quantification of Myc was recently reported for Myc-inducible P493-6 cells. It was found that before induction there were 13,000 molecules of Myc per cell and 77,000 and 362,000 molecules per cell after 1 or 24 h of induction. Taking into account the estimated volume of a mammalian cell nucleus of 4 × 10^-13^ liters, this means the concentration of Myc would start at 50 nM before induction and reach 1.5 μM after 24 h induction. If free DNA was available, Myc-Max should be bound before and after induction since the K_d_ for even low affinity sites is 20 nM. An analysis of DNase I hypersensitivity, FAIRE, and occupancy of DNA binding transcription factors from ENCODE data in five human cell lines led to the estimation that about 3% of the genome is relatively free of histones and available for binding of factors [48]. This would be about 180,000,000 bp considering there are two genomes per cell. This means that even at the highest concentration of Myc, there are 500 bp of available DNA per Myc molecule and, therefore, all Myc should be bound to DNA. Our *in vitro* binding data demonstrates that the top 16 8-mers cover the top 65% of the normalized occupancy (see Figure 4). One of these 8-mers should occur on average only every 4,000 bp. Therefore, most of the Myc should be bound to lower affinity sites and this is borne out by our bioinformatic analyses. It has been estimated that there are about 180,000 Pol II molecules engaged in transcription in the average HeLa nucleus and it is likely significant that the level of Myc in a high expressing cell is similar, since Myc correlates highly with the position of engaged Pol II. Sites of occupancy of Pol II and Myc rise concomitantly during induction of Myc in P493-6 cells (Additional file 1: Figure S4), further connecting Myc and promoter proximal paused Pol II [5].

The specific interaction(s) that bridges Myc with the transcription machinery is not clear, but Mediator is found in a similar position to Myc upstream of the promoter proximal paused Pol II. Like Mediator, Myc and Max peaked upstream of the TSS. Both the Myc and Mediator peaks extended into the downstream region occupied by Pol II. This could be due to crosslinking between the N-terminal transcription activation domain of Myc with Mediator, which has been shown to interact with both Pol II [49,50] and Myc [51], leading to indirect crosslinking through the polymerase to downstream DNA. Perhaps in an analogous manner, the N-terminal domain of KLF3 which interacts with other transcription factors but is not involved in DNA binding has been recently demonstrated to play a major role in genome occupancy [52]. Besides Mediator [51] and Pol II [53], Myc has been shown to associate with a number of factors including TRRAP, P-TEFb, chromatin remodeling machinery, DNA repair machinery, and other proteins involved in transcription and other processes [1,2,23,26,51,53].

Our results have clarified the DNA binding preferences of the Myc-Max heterodimer, widening the range of core binding sites bound specifically by Myc-Max and suggesting that non-specific interactions could also be significant. In examining binding to all possible 8-mers it became apparent that sequences flanking the core 6-mers were important. Changing the flanking sequences of the canonical E-box 6-mer from GCACGTGC to TCACGTGA resulted in a four- or five-fold reduction in Myc-Max and Max binding. A similar reduction has been previously noted for Pho4p but not Cbf1p, two yeast bHLH proteins [54,55]. Despite our expanded understanding of the relative interaction potentials of Myc-Max with all possible 8-mers, a strong correlation of Myc occupancy in cells to the underlying sequence could not be made.

How did it become so generally accepted that Myc-Max functioned as a specific DNA binding factor? As shown here and from earlier studies [9,11-13], Myc-Max does have a preference for certain sequences *in vitro*. Previously, these preferences were determined using EMSAs that routinely included unlabeled carrier DNA. Unfortunately, this leads to an inaccurate determination of the actual range of affinities for different sequences. Many reporter assays have been performed where the presence of a CACGTG E-box sequence enhances the expression of the reporter (for example, [56,57]). In almost all such experiments, only small (2-fold or less) effects are found and sequence-independent effects of Myc are normalized away. The small effects seen are consistent with our model in which having high affinity sites close to the promoter could fine tune the recruitment of Myc. Another issue arises from the misinterpretation of results obtained by programs, such as Multiple Em for Motif Elicitation (MEME) [58], which discover sequence motifs that are significantly enriched in a population of DNA sequences. However, identification of a motif (with a corresponding low *P* value) does not mean that sites matching the motif are present in all of the DNA sequences analyzed. In fact, very significant enrichment for a particular motif can be observed even when only a very small fraction of the analyzed sequences contain the motif. A second problem arises because of the length of the sequences analyzed and the resolution of actual binding site. Reasonable quality ChIP-Seq datasets like those analyzed here can determine individual binding sites to within 25 to 50 bps. If the fragments analyzed by a motif discovery algorithm are more than about 100 bp (as they usually are), a particular motif can be present in the sequence analyzed but not be bound (for example, see Figure 1B).

The model we propose for Myc might be applicable to other transcription factors. Significant overlap of occupancy between Myc and other factors such as AP-1 and AP-2 can be observed and two recent ENCODE reports showed co-association of many transcription factors binding to ‘surprisingly plastic’ DNA sequences [43,59]. Indeed, our results suggest that it may be time to revisit the significance of sequence-specific binding for many transcription factors in higher eukaryotes. The *E. coli* lac repressor paradigm [60], in which occupancy of a specific site on a 4 million bp genome is driven by a seven order of magnitude difference between specific and non-specific binding, may not apply to certain human transcription factors. The generally repressive structure of chromatin in eukaryotes can mask most non-specific and specific sites thereby reducing the complexity of DNA that would otherwise be available for binding [61]. In addition, nucleosomes would also obstruct 1-D sliding, one of the important mechanisms used by lac repressor to locate specific sites [62]. The human genome encodes about a thousand DNA binding transcription factors with a wide range of sequence specificity. At one extreme, CTCF is almost always bound to one of several sequence motifs, as determined by differential involvement of its 11 Zinc fingers [63]. TBP on the other hand is found at all promoters regardless of the presence of the TATA sequence it recognizes due to interaction with the transcription machinery [64]. The metazoan genomic landscape may be too complex for all factors to rely solely on sequence specificity for occupancy and function.

## Conclusions

We combined an extensive determination of the DNA binding properties of Myc-Max with genome-wide occupancies of Myc, Max, and Pol II and conclude that the affinity of Myc-Max for specific DNA sequences cannot be the main determinant of Myc genomic occupancy in cells. Our results indicate that the range in affinities *in vitro* for different sequences covers only a little over two orders of magnitude (K_d_ = 10^-8^ to 10^-10^ M). Even taking into account the fact that much of the human genome is not accessible due to chromatin structure, we found that Myc occupancy was not well correlated with affinity for underlying DNA in accessible regions. We found instead a strong correlation of Myc (and Max) occupancy with that of Pol II and on average Myc was located about 100 bp upstream of the promoter proximal paused Pol II. We propose that Myc is a general factor brought to promoters predominately by protein-protein interactions and like TBP, its recruitment to promoters does not strictly require sequence specific binding.

## Materials and methods

### ChIP-Seq datasets

The alignment files of HeLa, GM12878, and K562 cell lines were downloaded from human ENCODE Project at UCSC [65]. The raw sequence files of H128, H2171, MM1S, P493, and U87 cell lines were obtained from GSE36354. These raw sequences were aligned using ELAND to NCBI Build 36.1 (UCSC hg18) of the human genome. Only sequences that mapped uniquely to the genome with zero or one mismatch were used for further analysis. When multiple sequences mapped to the same genomic position, a maximum of two reads mapping to the same position were used. The sequenced reads were extended 200 bp to account for the size of sequenced fragments and then allocated into 25 base pair bins. The data from each bin were combined to generate the wiggle (WIG) files, which can be uploaded to UCSC genome browser. Max and Myc datasets from HeLa cells were further normalized for the total number of reads and the normalized Myc dataset was subtracted from normalized Max dataset to identify the genomic regions having extra Max compared to Myc.

### RefSeq gene list

The complete set of human RefSeq genes was downloaded from the UCSC table browser [66] on 1 January 2012. A custom annotated set of 20,886 RefSeq genes was generated by merging the TSSs from the same gene within 500 bases of each other, and removing the all TSSs within 1,000 bases of each other. This custom list was used for all analyses.

### Metagene analyses

The number of reads within 10,000 bases of the TSS of each RefSeq gene was tabulated without binning. The average value of the lowest 2,000 of 20,000 data points was subtracted from each position, and then data were normalized so that the area under each curve was equal. For Figure 2D, the data generated from the previous step were summed at each position using eight cell types from ENCODE (HeLa, GM12878, and K562) and GSE36354 (H128, H2171, MM1S, P493, and U87). Figure 2E was generated from GSE36354 (H2171, MM1S, P493, and U87).

### Generation of heatmaps

Heat maps were generated using the program R [67]. Genes were rank-ordered based on the sequence density for Pol II from -2 kb to +2 kb from the TSS. Using this order, base pair resolution sequence density for Pol II, Myc, Max, and CTCF for 20,886 genes was displayed without binning. The raw images were 21,000 × 4,000 pixels each and were adjusted identically using the gamma adjustment in Corel PhotoPaint (version X3) to allow visualization of the wide range of data (Figure 2B).

### Correlation of datasets

For Figure 2C, a total of 4 WIG tracks were generated using ENCODE data including Myc, Max, Pol II, and CTCF. The reads at each position were sorted in ascending order, and the cumulative percentage was calculated using R. The lowest 95% of the data points were subtracted as background since they represent mainly isolated single reads and the remaining reads were normalized to reads per million. The difference between two tracks was quantified as the sum of the absolute values of two tracks at each point. The correlation between two transcription factors was calculated within the range of 0 to 1, with a correlation of 1 indicating that the two tracks have a perfect match and 0 denoting total independence.

### Peak finding

A peak finding algorithm (ChIP-Seq Peak) [36] was applied to determine precise position and height of each significant peak of Myc, Max, and Pol II. The height is equal to the number of reads contributing to the peak not the highest value at the summit of the peak. The peak positions with heights at least 30 (about 10% of highest peak height) were used to retrieve the sequences within 50 bases of peak locations. To generate Max minus Myc peaks, the Myc and Max datasets were first normalized so that the total reads were 1 million and then subtracted from each other before the ChIP-Seq Peak algorithm was applied. The peaks identified were further annotated with the RefSeq genes and RNA expression levels (GSE23316) across the genome. A total of 351 genes that have a peak of extra Max within 250 bp of their TSSs were classified into three Gene Ontology (GO) categories [68]. The normalized HeLa Myc and Max ChIP-Seq datasets, the Max minus Myc dataset, and its associated ChIP-Seq Peak dataset are available on GEO (GSE43227).

### Expression and purification of proteins

Coding sequences of Myc (gi:29839758), Max^L^ (gi:21704261) and Max^S^ (gi:21704263) were amplified from HeLa cDNA and individually cloned into pET21a (C-terminal His-tag). All proteins were expressed in BL21 star *E. coli* after overnight induction of Max^L^ and Max^S^ at 18°C or 4 h induction of Myc at 37°C. Soluble Max proteins were purified over Ni-NTA and Mono Q for Max^S^ or Mono S for Max^L^ as described for Gdown1 [36]. Yields were approximately 20 mg per liter. Myc was purified over Ni-NTA and Mono Q in the presence of 7 M urea yielding 7 mg of Myc per liter. Both Myc-Max heterodimers were prepared by mixing equal moles of Myc and Max in 6 M urea followed by step dialysis against 4 M, 2 M, 1 M, and 0.5 M urea with 500 mM HGKEDP (25 mM HEPES, pH 7.6, 15% glycerol, indicated KCl, 0.1 mM EDTA, 1 mM DTT, and 0.1% of a solution of saturated PMSF in isopropanol) for 1.5 h each, then against 100 mM HGKEDP overnight. The renatured Myc-Max proteins were finally purified over Mono Q in HGKEDP. All protein samples were aliquotted and kept at -80°C. In the absence of urea, Myc was prone to aggregation, but Max and Myc-Max chromatographed cleanly and behaved well. Myc-Max heterodimers partially dissociated over time generating a small amount (<10%) of Max homodimers.

### EMSA

Binding reactions were 25 mM HEPES (pH 7.6), 100 mM KCl, 1 mM DTT, 0.1 mM EDTA, and 0.01% Triton. For reactions analyzed on silver stained gels, 200 ng of protein and 0, 0.1, 0.3, 1, or 3-fold molar excess of unlabeled dsDNA were used in each reaction. For the reactions using radiolabeled probes, each strand of the indicated probe was end-labeled with γ-^32^P-ATP and then annealed to generate dsDNA. Each reaction contained 0.01 nM labeled dsDNA and 0.01 pM, 0.1 pM, 1 pM, 0.01 nM, 0.1 nM, 1 nM, 10 nM, or 100 nM protein. For competition assays 0.01 nM labeled CACGTG probe, 1 nM of the indicated protein and the indicated amounts of non-labeled competitor DNA were used. After 30 min at room temperature, Ficoll (2% final) was added to each reaction and the samples were run on a 6% polyacrylamide gel in 0.5X Tris/glycine buffer at 10 mA for 2 h. The gels were then analyzed by silver staining or phosphorimaging.

### Protein-binding microarrays

Protein-binding microarray (PBM) experiments were performed as described previously [41]. Briefly, 4 x 44 K arrays (Agilent Technologies; AmadID 015681) containing the ‘all 10-mer’ universal PBM design were used. Arrays were incubated with a PBS buffer based protein mixture of 10 nM His-tagged Myc/Max heterodimer or Max homodimer, 2% milk, 200 ng/μL BSA, 50 ng/μL Salmon Testes DNA, and 0.02% TX-100. Bound protein was tagged with 10 ng/μL anti-His antibody conjugated to Alexa 488 (Qiagen; 35310) in PBS with 2% milk. The microarrays were scanned using a high-resolution GenePix 4400A scanner (Molecular Devices). Data were analyzed to obtain fluorescence intensities for all 8-mers, as described previously [41]. The raw median intensities and normalized *in vitro* occupancies for Myc-Max and Max-Max across all 8-mers are provided in Additional file 3: Table S2. Genome tracks were created by identifying all 8-mers in the human genome (NCBI build 36, hg18) that contain a core 6-mer that appeared in the top 200 8-mers (these 6-mers were CACGTG, CGCGTG, CACATG, CACGAG, CATGCG, AACGTG, CATGAG, CATATG, CATGCA, CATGCT, GTGCAC, and CTCGAG). Although only the central 6-mer was shown in the genome track, the *in vitro* occupancy scores were obtained by normalizing the observed intensity for each 8-mer to the intensity of the highest ranked 8-mer (in this case, CCACGTGG). The raw PBM data as well as the 8-mer intensities have been deposited in GEO (GSE58570) and the genome track for the top 12 6-mers has been deposited in GEO (GSE43227).

### Statistical analyses

Statistical analyses were performed in R. Fisher’s exact test (dhyper) was used to compare the frequency of CACGTG sites in Myc-occupied regions and in all accessible DNA (DNase I hypersensitive regions [43]). A similar analysis was performed using Pol II ChIP-Seq peaks instead of CACGTG sequences. In Figure 5A, *in vitro* Myc 8-mer occupancies were compared using the Wilcoxon rank-sum test (wilcox.test with the paired parameter set to false). A receiver operating characteristic (ROC) curve was used to assess the enrichment of sites with high *in vitro* 8-mer occupancies in the Myc ChIP-Seq peaks. A ROC curve is a plot of false positive rate (1-specificity) versus true positive rate (sensitivity) computed for different 8-mer occupancy cutoffs. Each point on the ROC curve corresponds to one cutoff. Sequences with 8-mer occupancy above or below the cutoff are predicted positives (that is, predicted to be bound by Myc *in vivo*) or predicted negatives (that is, predicted not bound by Myc *in vivo*), respectively. In Figure 5B, the correlation between Myc ChIP-Seq and *in vitro* occupancies was tested using Spearman’s rank correlation coefficient (cor.test with the method parameter set to Spearman).

## Additional files

Additional file 1:
**Supplementary Figures S1-S4.**
Additional file 2: Table S1. Top 1,487 peaks of extra Max. Additional file 3: Table S2. Raw median intensities and normalized *in vitro* occupancies for Myc-Max and Max-Max across all 8-mers.
