# Supplementary material for: Sequence specificity incompletely defines the genome-wide occupancy of Myc
Source: Genome Biol. 2014 Oct 7;15(10):482. doi: 10.1186/s13059-014-0482-3 (PMC4242493; doi:10.1186/s13059-014-0482-3)
Supplement: Additional file 1: — Supplementary Figures S1-S4. [file 13059_2014_482_MOESM1_ESM.pdf]

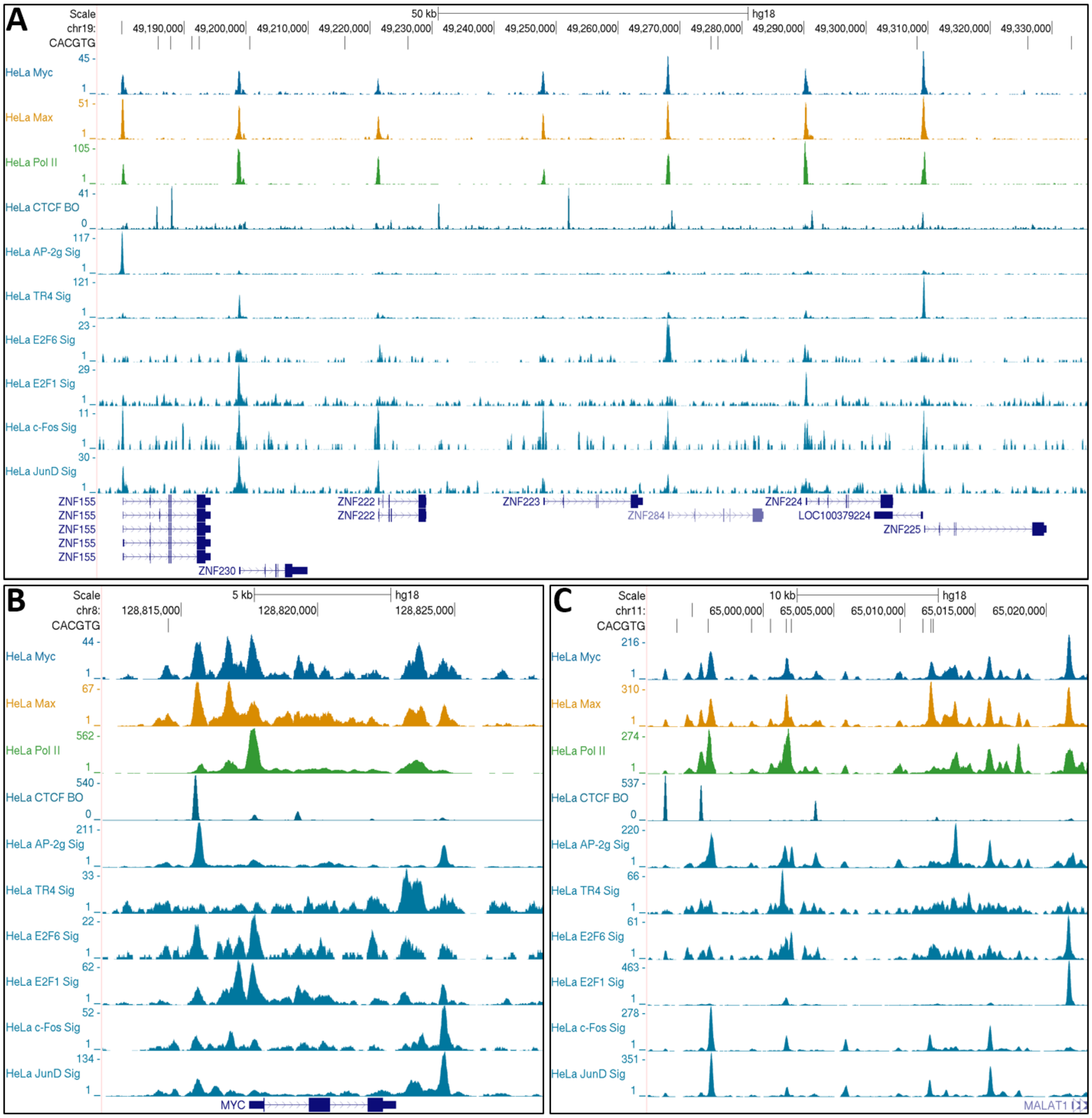

Figure S1. Examples of Pol II, Myc, Max, and additional factor occupancy. Genome browser tracks show occupancy determined by ChIP-Seq for Pol II, Myc, Max, CTCF, AP-2g, TR4, E2F6, E2F1, Fos and Jun over the indicated gene regions in HeLa cells. The positions of the canonical CACGTG E-boxes are indicated. Regions around **(A)** chromosome 19, **(B)** *MYC*, and **(C)** in the enhancer region upstream of *MALAT1* are shown.

	Myc	Max	Pol II	Fos	Jun	E2F1	CTCF
Myc		0.71	0.42	0.38	0.28	0.31	0.20
Max	0.71		0.41	0.39	0.27	0.30	0.19
Pol II	0.42	0.41		0.16	0.11	0.33	0.09
Fos	0.38	0.39	0.16		0.47	0.15	0.12
Jun	0.28	0.27	0.11	0.47		0.10	0.10
E2F1	0.31	0.30	0.33	0.15	0.10		0.09
CTCF	0.20	0.19	0.09	0.12	0.10	0.09	

Figure S2. Extended correlation analysis. HeLa ChIP-Seq datasets for the indicated factors were compared as described in the Methods section and in the text. A linear color scale was used and self-correlations were removed.

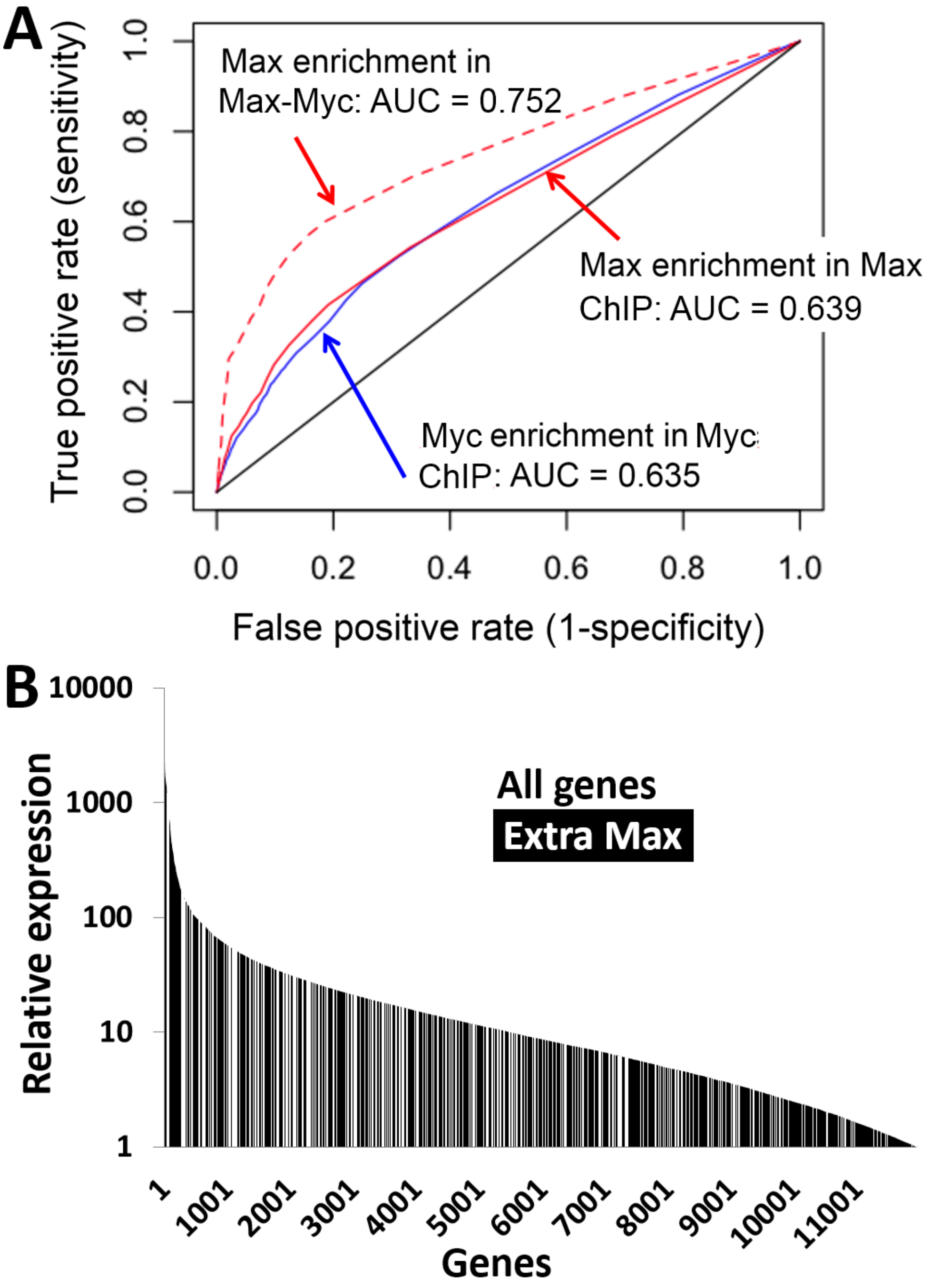

Figure S3. Analysis of peaks of extra Max. (A) Enrichment of 8-mers with high in vitro occupancy in the Myc, Max, and Max-Myc ChIP-Seq peaks. ROC curves were generated as in Figure 5*A*. The blue curve shows the enrichment of 8-mers with high Myc in vitro occupancy in the top 30,000 Myc ChIP-Seq peaks. The red curves show the enrichment of 8-mers with high Max in vitro occupancy in the top 30,000 Max ChIP-Seq peaks (full line) or the top 5,000 Max-Myc ChIP-Seq peaks (dotted line). The areas under the ROC curves (AUC) are shown. **(B)** Comparison of expression of genes with extra Max to expression of most genes. The top 12,000 genes based on RPKM values from the GSE23316 HeLa RNA-Seq dataset were rank ordered and their values graphed using an area plot. The 351 genes containing extra Max within 250 bp of their TSSs are displayed as white columns.

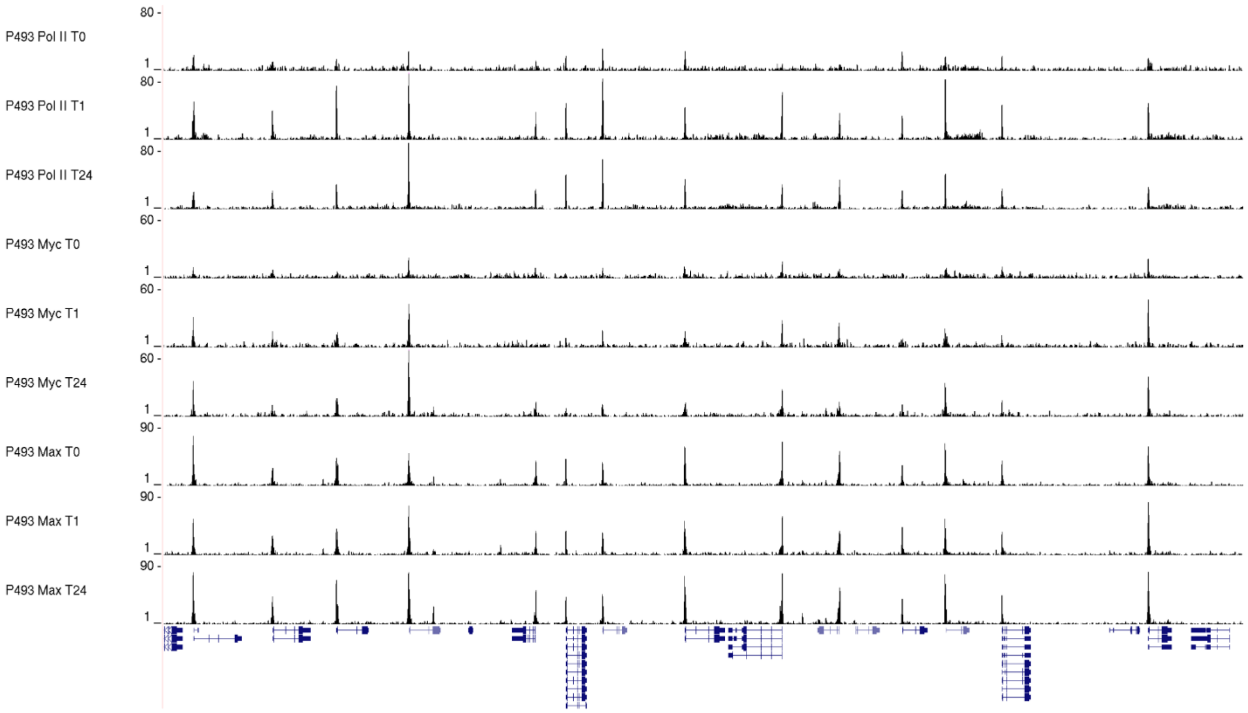

Figure S4. Induction of Myc in P493-6 cells correlates with increased Pol II occupancy. Data (GSE36354) are from Lin et al. Cell 2012. Tracks show occupancy of Pol II, Myc, and Max in a region of chromosome 19 (chr19:62,556,736-62,917,599). T0 is prior to induction of Myc and T1 and T24 are 1 and 24 hours after induction.
